# Polyorchidism: An incidental finding

**DOI:** 10.4103/0974-1208.57233

**Published:** 2009

**Authors:** Jyotsna Sen, Shalini Agarwal, Satish Prakash

**Affiliations:** Department of Radiodiagnosis, Pt. B.D. Sharma, PGIMS, Rohtak, Haryana - 124 001, India

Sir,

Only approximately 100 cases of polyorchidism have been reported in the literature since it was first described by Ahlfeld in 1880.[1] The importance of this condition lies in the fact that it can be confused with a mass lesion. We report one such case.

**Figure 1:**
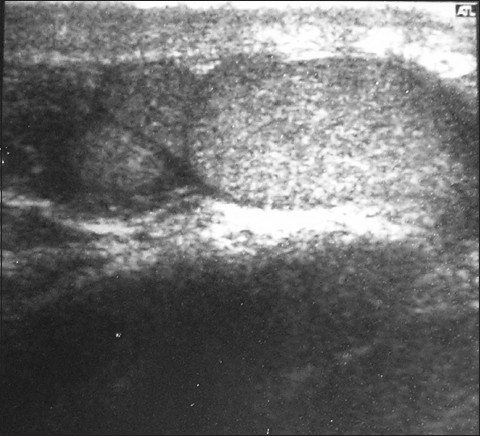
Longitudinal ultrasonographic scan of the right testis shows supernumerary testes at the lower pole

**Figure 2:**
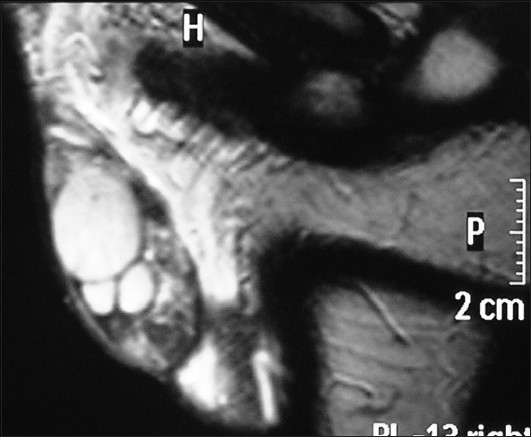
Sagittal T2-weighted scan of the right hemiscrotum with supernumerary testis at the lower pole

A 23-year-old male presented to the outpatient department because of vague heaviness in his inguinoscrotal region. No significant past medical history could be elicited. Physical examination revealed a discrete, ovoid, mobile and nontender lump at the lower pole of the right testis. He was then referred for ultrasonographic examination, which revealed three mass lesions on the right side of similar echogenicity [Figure 1]. The largest was the normal right testis measuring 2.6 × 1.3 cm whereas the other two masses were considered to be the supernumerary testis, which were located at the lower pole of the testis. They measured 1.1 × 1.0 cm and 1.1 × 0.7 cm, respectively. There was only one epididymis and vas deferens draining the normal testis. The testis and the epididymis on the left side were normal. The left testis measured 4.1 × 1.8 cm. Findings were confirmed on magnetic resonance imaging [Figure 2]. On urine examination, there was evidence of urinary tract infection. The patient was put on appropriate treatment for the same and showed a marked improvement in symptoms at follow-up 1 week later.

Triorchidism is the most common presentation, with only 75% of the supernumerary testes located in the scrotum. Approximately 50% of the cases are seen between the ages of 15 and 25 years. A prevalence of left-sided lesions (in the ratio of approximately 3:1) has been observed. The majority of the patients present with painless groin mass or testicular masses. The collective incidence of cancer among the reported series is 6.2%.[2] Spermatogenesis in the supernumerary testis is normal in about 50% of the cases.[1]

On the basis of embryologic development, Leung, in 1988, classified polyorchidism into four types whereas Singer *et al*. in 1992 proposed a classification based on the reproductive potential.[1] Thereafter, Mastroeni *et al*.[3] proposed a functional classification in 1997 based on embryologic development.

On imaging, they appear similar to the normal testis. Current treatment is conservative in the absence of complicating conditions like torsion, cryptorchism and malignancy. However, Danrad *et al*. have suggested a regular and frequent follow-up (3-6 months) with ultrasonography in case the supernumerary testis has its own epididymis. Otherwise, it may be prudent to remove it in view of the increased risk of malignancy.[4]
